# Porcelain Crown Work

**Published:** 1901-09-15

**Authors:** H. J. Goslee

**Affiliations:** Chicago, Ills.


### PORCELAIN CROWN WORK.

BY DR. H. J. GOSLEE, CHICAGO, ILLS.

Demonstrated at Tri-State Dental Meeting, Indianapolis, June, 1901.

In crowning the six anterior teeth the very objectionable feature of a metal backing extending to the incisal edge, which completely deprives the facing of its translucency, is overcome; as is also the occurrence of those dark blue lines along the joints between facing and backing due to the penetration of secretions, which objectionable and unhygienic condition is so often noticeable. Together with these advantages do we also reduce to a minimum the possibility of the facing becoming fractured in the mouth, because where the facing and backing are used the porcelain is retained to its backing simply by means of the attachment pins, while in the porcelain baked crown the facing is retained not only by the pins, but also by the etching or fusing of the "body" over the entire surface. This combined attachment makes it practically impossible to have the facing break away without all of the porcelain coming with it, which seldom occurs. Hence in point of relative strength there is no comparison, aside also from avoiding the display of any metal.

The bicuspids are particularly ideal indications for the porcelain crown, because of the simplicity in their construction and the difficulty of making any other style that would not entail much more effort and show more or less gold, and because of the frequency with which we here see the unsightly gold crown, which practice, in the face of our present opportunities for concealing the artificial, I now regard as almost criminal.

### ROOT-PREPARATION.

The requirements here are similar to those for any style of dowel crown with a band, excepting that all roots must be cut shorter because of the necessity for making as much space as possible for the porcelain, thus attaining the greatest degree of strength in the finished crown.

In sacrificing the remaining portions of the natural crown, however, they should first only be cut down to within about one-sixteenth of an inch of the cervix or gingivæ until the remaining ledge of enamel has been removed, the measurement taken, and the band fitted; each of these steps then being greatly facilitated by having this surplus or projecting end of the root to work upon, after which it may be cut down on a line with the gingivæ.

In dismissing the patient, however, after finishing this portion of the operation, the precaution should always be observed of packing temporary stopping into the canal and allowing it to cover the end of the root, so as to prevent the tissue from crawling over the exposed edge during the interim, as this often interferes with the permanent setting of the crown when completed. Where the crown is for an anterior tooth it is a good and most satisfactory practice to make a temporary one to be worn while the permanent one is being finished. This consumes but a few moments' time, or only long enough to select a facing, grind it to fit approximately fit a german silver post to the canal, and soft solder it to the facing ; then mount with temporary stopping. The gum is kept back nicely, and the patient is not subjected to the usual temporary disfigurement.

### BANDS.

The fitting of the band is, of course, very important, but is rendered more or less easy by leaving the root as described until this step has been accomplished, as it serves to comform it to the proper shape and guides it into place. In pressing it into position it should be so trimmed as to meet the gum line evenly at all sides before passing under it, and should be as narrow as possible to meet the requirements ; but being narrow, and in order that it may possess strength, no thinner than twenty-eight gauge pure platinum should be used.

If pure gold be used for solder, a lapped joint should always be made, which precludes the possibility of its opening under the influence of expansion, which precedes the shrinkage, in the subsequent baking. Where platinum solder is used the joints may be butted.

### CAP.

The metal used for the floor to the band which forms the cap should be preferably of thirty-two gauge platino-iridium, in order that the cap may possess every available strength sufficient to permanently retain its shape.

### POSTS.

In the preparation of canals for the reception of posts care should be exercised to avoid enlarging them any more than is absolutely necessary. The post should be proportionate in size with the root—sixteen gauge (B. & S.) for centrals, cuspids, and molars, and eighteen gauge for laterals, bicuspids, and all of the lower anterior; and should be of platino-iridium, in order to be stiff, rigid and unyielding. They should extend into the canal a distance nearly equal to the length of the crown, in order to overcome the force of leverage. Should fit at least two walls closely, and in passing through the floor of the cap should be placed far enough to the lingual so that the surplus end may not interfere with the facing in its adjustment to proper relation with the cap. A square or triangular post is preferable, because of overcoming the tendency to rotate on conical roots, and for the reason that a wire drawn with sharp angles is more rigid than a round one.

### FACINGS.

The selection of facings should be made very carefully, so that as little grinding as possible may be necessary. In point of color a very slight tendency to the darker is advisable, because they sometimes bleach just a trifle in baking. The neck of the facing should be ground very thin so as to overlap the cap, for the reason that it is desirable to bring the neck in close contact with the gum and to cover the labial or buccal surface of the band with porcelain to avoid or prevent its showing in case of recession. If this joint between the edge of facing and the cap be smooth and continuous it will be found that the tissues take very kindly to it, and seldom present those evidences of irritation and inflammation so often apparent. The main object in overlapping the facing is to securely anchor the body in the joint when fused, for in this connection it must be remembered that there is absolutely no physical union between the porcelain and platinum; and when it is desirable to fuse porcelain over the surface of platinum, to be retained permanently, mechanical, means, such as etching or roughing, etc., must be resorted to.

The facing must always be soldered to the cap in order to secure the combined strength of this attachment together with that of the porcelain in fusing. In the six anterior teeth the pins must come in contact with the surplus end of post at a point as close to the floor as convenient, in order that they may allow as much room as possible for the body and in no way interfere with the contouring of the lingual surface of the crown. In some instances they may even brought into direct contact with the floor and soldered.

In the bicuspids the same general details apply, together with an additional important consideration, which is that while the porcelain must be securely anchored to the metal construction, it must be also supported against stress and cleavage. Hence it is here necessary to add to the metal parts a support for the mass of porcelain which is to form the lingual cusp. This is obtained by a simple vertical bar soldered to the center of the lingual portion of the cap, extending about one-half way to the occlusal edge of the facing; or it may be an extension of the lingual post if two posts are used. The end of this extension, however, must always be nicely rounded, or, acting as a wedge under applied force, it will prove an element of weakness instead of strength.

SOLDERING.

In the attachment of the various parts of the metal construction for single crown-work absolute contact of the parts to be united is essential, and when the facing is being soldered the entire lingual surface should be freely exposed in the investment. Pure gold can then be used with every assurance of a strong union, even after baking. Only enough, however, should be used to make the joint, and it should be well and thoroughly fused that all surplus may become absorbed by the platinum, so as not to remain in globular form to melt and become absorbed under the porcelain during the baking, which invariably causes a porosity.

Platinum and gold solders in various percentages are being extensively used now with a view to uniting the joints with a solder which is not melted at the fusing point of the body. Such is an advantage in bridge-work where the shrinkage of porcelain materially affects the relation of the parts, but is not necessary in single crown-work. After the soldering has been completed the crown should be subjected to the acid bath, then washed clean in order that no particles of borax or investing material remain to interfere with the vitrification of the body. All sharp angles of metal should then be nicely rounded with a disk, as the body will not fuse smoothly over them, and they will prove an element of weakness.

I use the ordinary continuous gum body, which vitrifies at from 2200° to 2500° F., which, because of its superior quality and strength, is the only material I regard as possessing the necessary qualifications for general use. Before using it, however, it is necessary to put it in the mortar and pulverize it much finer, as it can not be nicely manipulated and carved until this has been done. When reduced to a finer consistence it works, carves, and fuses most beautifully, and no doubt will ultimately be prepared for use in this shape and designated as crown and bridge "body." At the present, however, these bodies do not come to us in any variety of colors, so it becomes necessary to depend entirely upon the facings for such.

Prior to building up and carving the work should be fitted on a fire-clay support which will hold it securely and firmly and sustain a perpendicular position, as this is very necessary and can be more easily done before the porcelain is added than afterward, when it should be handled but little.

